# Bioconversion From Docosahexaenoic Acid to Eicosapentaenoic Acid in the Marine Bacterium *Shewanella livingstonensis* Ac10

**DOI:** 10.3389/fmicb.2020.01104

**Published:** 2020-05-26

**Authors:** Takuya Ogawa, Kazuki Hirose, Yustina Yusuf, Jun Kawamoto, Tatsuo Kurihara

**Affiliations:** Molecular Microbial Science, Institute for Chemical Research, Kyoto University, Kyoto, Japan

**Keywords:** eicosapentaenoic acid, docosahexaenoic acid, marine bacteria, bioconversion, β-oxidation

## Abstract

Eicosapentaenoic acid (EPA) and docosahexaenoic acid (DHA), which belong to the same class of long chain ω-3 polyunsaturated fatty acids (PUFAs), are present in marine γ-proteobacteria. In contrast to their *de novo* biosynthesis that has been intensively studied, their metabolic fates remain largely unknown. Detailed information regarding bacterial ω-3 PUFA metabolism would be beneficial for understanding the physiological roles of EPA/DHA as well as the industrial production of EPA, DHA, and other PUFAs. Our previous studies revealed that the EPA-producing marine bacterium *Shewanella livingstonensis* Ac10 produces EPA from exogenous DHA independently of *de novo* EPA biosynthesis, indicating the presence of an unidentified metabolic pathway that converts DHA into EPA. In this study, we attempted to reveal the molecular basis for the bioconversion through both *in vivo* and *in vitro* analyses. Mutagenesis experiments showed that the gene disruption of *fadH*, which encodes an auxiliary β-oxidation enzyme 2,4-dienoyl-CoA reductase, impaired EPA production under DHA-supplemented conditions, and the estimated conversion rate decreased by 86% compared to that of the parent strain. We also found that the recombinant FadH had reductase activity toward the 2,4-dienoyl-CoA derivative of DHA, whereas the intermediate did not undergo β-oxidation in the absence of the FadH protein. These results indicate that a typical β-oxidation pathway is responsible for the conversion. Furthermore, we assessed whether DHA can act as a substitute for EPA by using an EPA-less and conversion-deficient mutant. The cold-sensitive phenotype of the mutant, which is caused by the lack of EPA, was suppressed by supplementation with EPA, whereas the DHA-supplementation suppressed it to a lesser extent. Therefore, DHA can partly substitute for, but is not biologically equivalent to, EPA in *S. livingstonensis* Ac10.

## Introduction

Various marine γ-proteobacteria produce ω-3 long chain polyunsaturated fatty acids (PUFAs). Among them, a group of bacteria, which includes species *Shewanella pneumatophori* SCRC-2738 and *Photobacterium profundum* SS9, mainly produces (all-*Z*) eicosa-5,8,11,14,17-pentaenoic acid (EPA) (Yazawa, 1996; Nogi et al., 1998). Another group includes other species, such as *Colwellia psychrerythraea* H34 and *Moritella marina* MP-1, which mainly produce (all-*Z*) docosa-4,7,10,13,16,19-hexaenoic acid (DHA) (DeLong and Yayanos, 1986; Hashimoto et al., 2015). As are found in and beneficial to a broad range of organisms, including mammals, nematodes, fungi, and algae, EPA and DHA are physiologically important for marine bacteria and have been proven to render the cells resistant to hostile environments, such as low temperatures and high hydrostatic pressures, and to hydrogen peroxide-induced oxidative stress (Nishida et al., 2006; Kawamoto et al., 2009, 2011). Bacterial PUFA production was previously believed to be limited to marine bacteria, but has since also been reported in recent years in members of δ-proteobacteria retrieved from soil samples (Garcia et al., 2011, 2016).

The past two decades of research have uncovered a bacterial biosynthetic route for EPA/DHA that is unique in employing polyketide synthase-like multifunctional enzymes. The enzymes catalyze repeated cycles of decarboxylative condensation of malonyl-CoA with a growing fatty acyl group, which originates from the acetyl group, and C=C bond-forming dehydration, and eventually release either EPA or DHA as the final product (Yoshida et al., 2016). The genes for PUFA synthesis are spread widely among the above-mentioned bacteria, but their gene products are composed of different catalytic domains and provide different final products, such as EPA, DHA, or other olefinic lipids (Orikasa et al., 2009; Shulse and Allen, 2011; Hayashi et al., 2019). The bacterial PUFA biosynthesis system differs from the eukaryotic system wherein PUFAs are produced from a pre-existing fatty acid, e.g., α-linolenic acid [namely (all-*Z*) octadeca-9,12,15-trienoic acid], by the alternating actions of multiple elongases and desaturases (Metherel and Bazinet, 2019). In bacteria, EPA and DHA are generally introduced into the *sn*-2 position of glycerophospholipids and constitute the lipid bilayer of cellular membranes. The membrane-bound enzyme lysophosphatidic acid acyltransferase (PlsC) is responsible for the acylation step (Cho et al., 2012; Ogawa et al., 2018). However, in contrast to the biosynthetic pathways, the metabolic fates of EPA and DHA, such as degradation and conversion in bacteria, have not been well studied.

*Shewanella livingstonensis* Ac10 is a psychrotrophic bacterium originally isolated from Antarctic seawater and produces EPA, but not DHA, in response to low temperatures (Kawamoto et al., 2009). Like other EPA- and DHA-producing bacteria, *S. livingstonensis* Ac10 utilizes polyketide synthase-like enzymes to produce EPA *de novo*, and mutants that lack the genes coding for these enzymes produce no EPA, indicating that the polyketide synthase pathway is solely responsible for *de novo* EPA biosynthesis in this bacterium (Kawamoto et al., 2009). To investigate the physiological roles of EPA, we previously characterized the EPA-less mutant (ΔEPA) of *S. livingstonensis* Ac10. The mutant showed cold-sensitive phenotypes, such as growth retardation and aberrantly elongated cell shapes, when grown at 4°C, while its phenotypes were similar to those of the wild-type when grown at 18°C (Kawamoto et al., 2009). The defects were suppressed by supplementation with a synthetic phospholipid (PL) that has EPA as its acyl chain (Sato et al., 2008; Kawamoto et al., 2009) and with free EPA (Tokunaga et al., 2017). Notably, a synthetic DHA-containing PL also suppressed the growth defects despite the absence of DHA in a wild-type cell of *S. livingstonensis* Ac10 (Sato et al., 2008). However, whether DHA (moiety) was beneficial to the bacterium remained ambiguous because PL analysis showed that the ΔEPA cells unexpectedly produced EPA upon supplementation with DHA-containing PL. This finding indicated the presence of an unidentified metabolic route that generates EPA from exogenous DHA, thereby bypassing the loss of *de novo* EPA biosynthesis. Such a pathway has also been proposed for *Escherichia coli* (Watanabe et al., 1994), although the underlying mechanism is yet to be elucidated.

In this study, we attempted to reveal the conversion mechanism by both *in vivo* mutagenesis experiments and *in vitro* enzymatic assays and found that it is mediated through a typical β-oxidation pathway that involves an auxiliary β-oxidation enzyme 2,4-dienoyl-CoA reductase. Furthermore, as we obtained a mutant that no longer metabolizes DHA to EPA, we reassessed the biological equivalency of EPA and DHA by using an EPA-less and conversion-deficient mutant.

## Materials and Methods

### Reagents

EPA and DHA were purchased from Nacalai Tesque (Kyoto, Japan), coenzyme A (CoA) from Oriental Yeast (Tokyo, Japan), and acyl-CoA oxidase (ACOX) from Asahi KASEI (Tokyo, Japan). All other chemicals used were of analytical grade.

### Bacterial Strain and Cultivation

The ΔEPA strain, which was derived from the Δ*pyrF* strain of *S. livingstonensis* Ac10 by deleting the *orf5* gene (Ito et al., 2016), and its derivatives were cultivated at 4°C in Luria-Bertani (LB) medium that was, when indicated, supplemented with 50 μg/mL kanamycin (Km), 50 μg/mL chloramphenicol (Cm), 50 μg/mL rifampicin (Rf), and/or 40 μg/mL uracil (Ura). EPA and DHA were dissolved in ethanol and added to LB medium at a final concentration of 32 μM (or 128 μM when indicated). The transformants of *E. coli* S17-1/λ*pir* were cultured in LB medium supplemented with 50 μg/mL Km or Cm, and those of *E. coli* BL21(DE3) were cultured in LB medium supplemented with 100 μg/mL ampicillin.

Bacterial growth at 6°C in the presence and absence of 128 μM EPA or DHA was monitored by measuring the optical density at 600 nm (OD_600_) using a rocking incubator TVS062CA (ADVANTEC, Tokyo, Japan). The cell morphology at OD_600_ of about 1.0 was observed using a BX40F4 trinocular microscope (OLYMPUS, Tokyo, Japan) equipped with a Moticam 2500 digital microscope (Shimadzu Rika, Tokyo, Japan). The software ImageJ version 1.52q^1^ was used to quantify the longitudinal cell length.

### Mutagenesis

Single-crossover homologous recombination-based mutagenesis was carried out to disrupt six putative *fadH* genes, i.e., *sl_221*, *sl_453*, *sl_1274*, *sl_1301*, *sl_1351*, and *sl_1692* (Accession numbers: LC528929, LC528930, LC528931, LC528932, LC528933, and LC528934, respectively). Approximately 500 bp internal regions of the genes were amplified by PCR using Q5 DNA polymerase (New England Biolabs, Ipswich, MA, United States), the genome of *S. livingstonensis* Ac10 as the template, and the primer pairs listed in Table 1. The linearized pKNOCK-Km was prepared by inverse PCR using the primer pairs listed in Table 1. Each of the amplified gene fragments was inserted into the *Sma*I site of pKNOCK-Km using a NEBuilder HiFi DNA Assembly kit (New England Biolabs), following the manufacturer’s instructions. The competent cells of *E. coli* S17-1/λ*pir* were transformed with the resulting plasmids, which were thereafter screened on a Km-supplemented LB agar plate. After the transformants and ΔEPA strain of *S. livingstonensis* Ac10 were cultivated in LB medium to OD_600_ of about 1.0, 100 μL of each culture was mixed and spread onto an LB agar plate. The bacterial lawns that formed after overnight incubation at 18°C were collected and suspended in 1 mL of LB medium, and 200 μL of the cell suspension was spread onto the LB agar plate supplemented with Km and Rf. The single colonies that formed on the selection plate were screened by colony PCR using the diagnostic primers listed in Table 1 to obtain six *fadH*-disrupted mutants, which are hereafter designated as, for example, ΔEPA/*sl_1351*.

**TABLE 1:** Primers used in this study.

Target DNA	DNA sequences of forward (Fw) andreverse (Rv) primers
**For mutagenesis**	
pKNOCK-Km	Fw, 5′-GGGCTGCAGGAATTCGATATCAAGC-3′
	Rv, 5′-GGGGGATCCACTAGTTCTAGAGCG-3′
*sl_221*	Fw, 5′-CTAGAACTAGTGGATCCCCCCTACCCAAATACCCCTGGTTTATTTACTCC-3′
	Rv, 5′-ATATCGAATTCCTGCAGCCCACTCAGGTAACAAGTAGTCAAACACGTCAC-3′
*sl_453*	Fw, 5′-CTAGAACTAGTGGATCCCCCATCATTACCATAATTTAGCCCAGTCTGGTG-3′
	Rv, 5′-ATATCGAATTCCTGCAGCCCTTACTGACTATTTCGAGCAATATTCTGGCG-3′
*sl_1274*	Fw, 5′-CTAGAACTAGTGGATCCCCCGAAGTTGCAGCATATTACCGCAGAC-3′
	Rv, 5′-ATATCGAATTCCTGCAGCCCACTATTTCAACACCAAAGCGAGTACGATT-3′
*sl_1301*	Fw, 5′-CTAGAACTAGTGGATCCCCCTGAATATTATGCTCAACGCGCTTCAGC-3′
	Rv, 5′-ATATCGAATTCCTGCAGCCCACCACCATATTCGTCAATACGATTATTCG-3′
*sl_1351*	Fw, 5′-CTAGAACTAGTGGATCCCCCAAGCTTTTCTTGGCAGGTGGGTA-3′
	Rv, 5′-ATATCGAATTCCTGCAGCCCACCGGTATTAATAATACTTACGGCTGCTTG-3′
*sl_1692*	Fw, 5′-CTAGAACTAGTGGATCCCCCACCAGACGATGCATTGTTCAACTTG-3′
	Rv, 5′-ATATCGAATTCCTGCAGCCCATCGGCACCACATTTAAGTCTTACTTGC-3′
**For mutant selection**	
*sl_221*	Fw, 5′-ACATTCTTGTACGATTAGCGCTAAATAAGC-3′
*sl_453*	Fw, 5′-TATGCTGGTTAGGTTTCAGTATGTTGCGTG-3′
*sl_1274*	Fw, 5′-TCTGTTAATGGCGTCATCCCAGGC-3′
*sl_1301*	Fw, 5′-ACGTATACTTCACTTGGCAATGTAACC-3′
*sl_1351*	Fw, 5′-TGCTGATTGATCTTCGACGACAATTTCAT-3′
*sl_1692*	Fw, 5′-AGCTCATTTGGTATTCCAGATGGTTATGT-3′
pKNOCK-Km	Rv, 5′-ACGTGTTCCGCTTCCTTTAGCA-3′
**For complementation**	
pJRD-Cm^r^	Fw, 5′-TAGTATAGTCTATAGTCCGTGG-3′
	Rv, 5′-CGTAATCCATGGATCAAGAG-3′
*sl_1351* with its promoter	Fw, 5′-GATCCATGGATTACGACGTTGATGATGAACTTACGGATACC-3′
	Rv, 5′-CTATAGACTATACTATTAAATACTCATCGCAAGTTCTGCACC-3′
cloning site	Fw, 5′-CCAGCTCTTTCTGCAGTTCATTC-3′
	Rv, 5′-CTGGATTTCACTGATGAGAATATCGTCG-3′
**For heterologous expression in *E. coli***	
pET15b	Fw, 5′-ATGGCTGCCGCGCGGCAC-3′
	Rv, 5′-GGATCCGGCTGCTAACAAAG-3′
*sl_1351*	Fw, 5′-GTGCCGCGCGGCAGCCATATGTCGTTTCCACACTTATTAGAACCT-3′
	Rv, 5′-CTTTGTTAGCAGCCGGATCCTTAAATACTCATCGCAAGTTCTGCACC-3′
*sl_3390*	Fw, 5′-GTGCCGCGCGGCAGCCATATGATCTACCAAAGCCCTACCATTCAG-3′
	Rv, 5′-CTTTGTTAGCAGCCGGATCCTTAGGCTTGGTAGTAACTACCATTGTTGG-3′

For complementation, the *sl_1351* gene together with its 249 bp-upstream region (Accession number: LC528933), which contains a putative promoter predicted on the Neural Network Promoter Prediction website^2^, and pJRD-Cm^r^ (Toyotake et al., 2018) were PCR-amplified using the primer pairs listed in Table 1. The two PCR products were fused using an In-Fusion cloning kit (Takara Bio, Shiga, Japan) to obtain pJRD-sl_1351. *E. coli* S17-1/λ*pir* transformed with the plasmid and the ΔEPA/*sl_1351* strain were cultivated in LB medium to OD_600_ of 0.2–0.3, co-cultured on an LB agar plate, then screened on an LB agar plate supplemented with Cm and Km. The mutant harboring pJRD-sl_1351 was selected by colony PCR using the diagnostic primers listed in Table 1.

### Electrospray Ionization (ESI)-Mass Spectrometry (MS) Analysis of PL Composition

The parent and mutant strains were cultivated in 5 mL of Ura-supplemented LB medium at 4°C. After the cells were grown to OD_600_ of about 1.0, they were harvested and subjected to Bligh and Dyer lipid extraction (Bligh and Dyer, 1959). The resulting lower (chloroform-methanol) phase was collected and dried under a stream of nitrogen gas, and the residual lipids were dissolved in methanol/acetonitrile (1:1) containing 0.1% triethylamine for ESI-MS analysis. The API3000 triple-quadrupole mass spectrometer (SCIEX, Ontario, Canada) equipped with an electrospray ion source was used to scan the total ions with the following parameters: polarity, negative; nebulizer gas, 8; curtain gas, 8; ion spray voltage, −4,200 V; declustering potential, −30 V; focusing potential, −200 V; and entrance potential, −10 V. In addition, EPA- and DHA-containing PLs were selectively scanned in the precursor ion mode with target *m/z* of 301.4 and 327.4, respectively, with the following parameters; collision energy, −45 eV, and collision cell exit potential, −15 V.

### Gas Chromatography (GC)-Coupled MS Analysis of Fatty Acid Composition

Fatty acid methyl esters (FAMEs) were prepared from the lipids extracted as described above following the methods reported previously (Ichihara and Fukubayashi, 2010). Briefly, the dried lipid extracts were dissolved in 0.2 mL of toluene, mixed with 1.5 mL of methanol and 0.3 mL of 8% HCl/methanol, and incubated at 45°C for 14 h. After cooling to ambient temperature, 1 mL of water and *n*-hexane, respectively, was added and the solution was vigorously shaken to extract FAMEs, followed by concentration to about 400 μL under a stream of nitrogen gas. The FAMEs were analyzed using a Clarus SQ 8C mass spectrometer interfaced with a Clarus 680 gas chromatograph (Perkin Elmer, Wellesley, MA, United States) equipped with a DB-1 capillary column (30 m × 0.25 mm, 0.25 μm; Agilent Technology, Santa Clara, CA, United States). The experiments were performed in triplicate, and the peak area for each FAME was divided by the sum of the peak areas for total FAMEs to calculate the fatty acid composition.

To estimate the conversion rate from DHA to EPA, 1 mL of bacterial culture was extracted via the Bligh-Dyer method, and the chloroform-methanol phase was dried, methyl-esterified, and analyzed by GC/MS, as described above. The experiments were performed in triplicate, and the peak area for EPA methyl ester was divided by the sum of the peak area for DHA and EPA methyl esters to calculate the conversion rate.

### Heterologous Expression and Purification of Sl_1351 and Sl_3390

The genes *sl_1351* and *sl_3390* (Accession numbers: LC528933 and LC528935, respectively) were PCR-amplified using the genome of *S. livingstonensis* Ac10 as the template and the primer pairs listed in Table 1, while the linearized pET15b was prepared by inverse PCR using the primer pair listed in Table 1. The respective genes were inserted into the *Nde*I-*Bam*HI site of pET15b using a NEBuilder HiFi DNA Assembly kit to obtain pET15b-sl_1351 and pET15b-sl_3390. *E. coli* BL21(DE3) transformed with pET15b-sl_1351 was cultured in 350 mL of ampicillin-supplemented LB medium at 37°C until OD_600_ reached 0.4, and was subsequently cultured at 18°C for a further 22 h. *E. coli* BL21(DE3) transformed with pET15b-sl_3390 was cultured in the same way, except the protein expression was induced with 0.5 mM isopropyl-β-D-1-thiogalactoside following the temperature down-shift. The cells were harvested by centrifugation and stored at −30°C for future use.

The collected cells (approximately 1 g of wet cells) of *E. coli* BL21(DE3) harboring pET15b-sl_1351 were suspended in 5 mL of binding buffer (20 mM sodium phosphate, pH 7.4, 500 mM NaCl, 20 mM imidazole, and 0.1 mM dithiothreitol) and disrupted by sonication. After the lysate was clarified by centrifugation (20,000 *g* for 30 min at 4°C), the supernatant was recovered and loaded to a HisTrap FF 1 ml column (GE healthcare, Chicago, IL, United States) pre-equilibrated with the binding buffer. The column was washed with 10 mL of wash buffer (20 mM sodium phosphate, pH 7.4, 500 mM NaCl, 60 mM imidazole, and 0.1 mM dithiothreitol) then eluted with 5 × 1 mL of elution buffer (20 mM sodium phosphate, pH 7.4, 500 mM NaCl, 500 mM imidazole, and 0.1 mM dithiothreitol). The eluates were pooled and subjected to ultrafiltration with an Amicon Ultra 50 kDa Cutoff Filter Unit (Merck, Darmstadt, Germany) to concentrate the recombinant proteins and dilute imidazole by repeated dilution-concentration. The protein profile was analyzed by SDS-PAGE (9% acrylamide), and protein content was measured via the Bradford method. Recombinant Sl_3390 was similarly purified, except buffers containing no dithiothreitol were used.

### *In vitro* Enzymatic Assays

Fatty acyl-CoAs were synthesized through the mixed-anhydride method, as described elsewhere (Ogawa et al., 2018). In the product analysis for Sl_1351, the 100 μL reaction mixture containing 0.1 mM DHA-CoA, 0.1 unit of ACOX, 1 μM purified Sl_1351, 1 μM purified Sl_3390, 0.2 mM NADPH, 1.0 mM *N*-2-hydroxyethylpiperazine-*N*′-2-ethanesulfonic acid (HEPES)-NaOH (pH 7.0), and 0.12 mM dithiothreitol was incubated at 20°C for 10 min. The reaction mixture was placed on ice to terminate the reaction and then desalted with an Oasis HLB 1 cc cartridge (Waters, Milford, MA, United States; Magnes et al., 2005). The cartridge was washed with 3 mL of acetonitrile and equilibrated with 2 mL of 1.0 mM HEPES-NaOH (pH 7.0), followed by sample loading. The cartridge was washed with 4 mL of water and finally eluted with 0.5 mL of acetonitrile/water (1:1) containing 15 mM ammonium hydroxide. The eluates were analyzed by ESI-MS with the following parameters: polarity, positive; nebulizer gas, 8; curtain gas, 8; ion spray voltage, 4,200 V; declustering potential, 65 V; focusing potential, 200 V; and entrance potential, 10 V.

In the product analysis for Sl_3390, the 100 μL reaction mixture containing 0.1 mM palmitoyl-CoA, 0.1 unit of ACOX, 1 μM purified Sl_3390, 0.2 mM NAD, and 1.0 mM HEPES-NaOH (pH 7.0) was incubated at 20°C for 10 min. The product was desalted and subjected to ESI-MS analysis, as described above.

## Results

### The Conversion of Exogenous DHA Into EPA in the ΔEPA Strain

**FIGURE 1:**
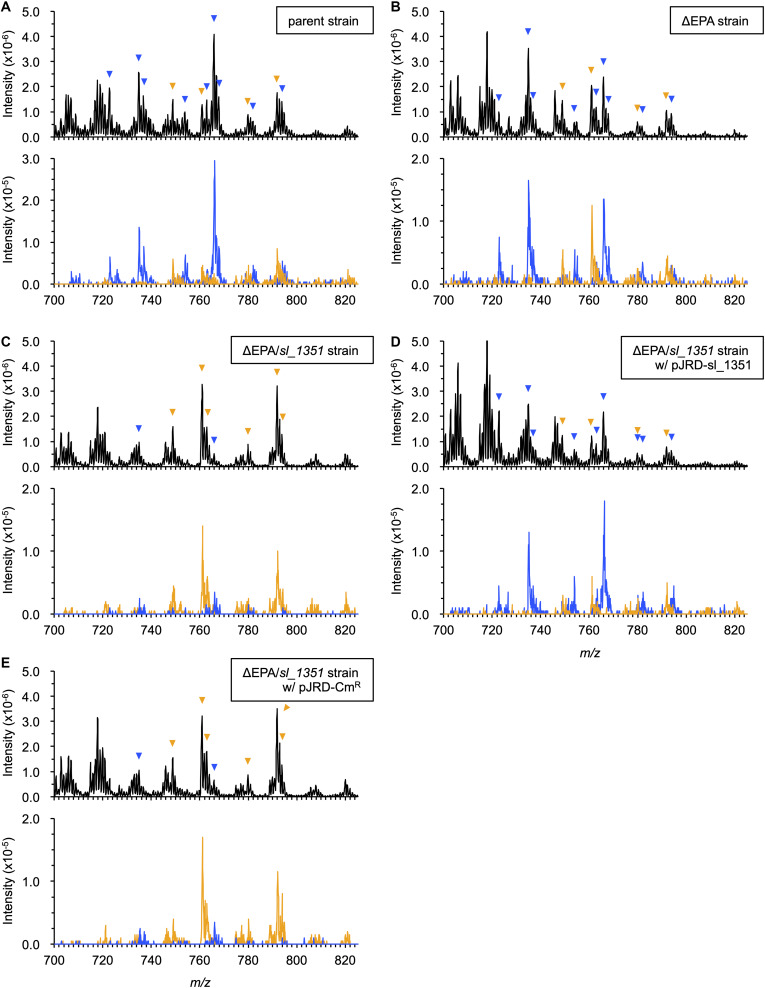
ESI-MS analyses of PL compositions. The PLs were extracted from the parent strain **(A)**, ΔEPA strain **(B)**, ΔEPA/*sl_1351* strain **(C)**, ΔEPA/*sl_1351* strain harboring pJRD-sl_1351 **(D)**, and ΔEPA/*sl_1351* strain harboring pJRD-Cm^r^ **(E)** grown in the presence of DHA. Three spectra of total ions (upper panel) and precursor ions for *m/z* of 301.4 and 327.4 (blue and orange traces on lower panel, respectively) are shown. The arrowheads indicate EPA- (blue) and DHA-containing PL ions (orange), of which predicted fatty acyl compositions are shown in Table 2.

In our previous study, the ΔEPA strain was supplemented with DHA as a form of the acyl group of a synthetic PL (Sato et al., 2008). As *S. livingstonensis* Ac10 can incorporate exogenous free EPA and utilize it to form membrane PLs (Tokunaga et al., 2017), we supplemented the ΔEPA strain with a free DHA in this study and analyzed its PL profiles by ESI-MS (Figure 1 and Supplementary Figure S1). In addition to a total ion scan, a precursor ion scan for ions of eicosapentaenoate (*m/z* 301.4) and docosahexaenoate (*m/z* 327.4) was performed to selectively detect EPA- and DHA-containing PLs. Without supplementation of DHA, the parent strain produced EPA-containing, but not DHA-containing, PLs (Supplementary Figure S1A and Table 2), whereas neither were detected in ΔEPA cells (Supplementary Figure S1B). On the other hand, when grown in the presence of DHA, the parent strain produced both EPA- and DHA-containing PLs (Figure 1A and Table 2), indicating that the bacterium utilized exogenous DHA to form membrane PLs. It was also noted that the ΔEPA strain produced EPA- as well as DHA-containing PLs in DHA-supplemented conditions (Figure 1B and Table 2). These data suggested that *S. livingstonensis* Ac10 could produce EPA from exogenous DHA.

**TABLE 2:** Representative *m/z* values of [M-H]^–^ ions of EPA- and DHA-containing PLs detected in ESI-MS analysis.

		Headgroup	
		Ethanolamine	Glycerol
Acyl chains	15:0/20:5	723.0	753.8
	16:1/20:5	735.1	766.0
	16:0/20:5	736.9	768.0
	17:1/20:5	748.9	780.2
	17:0/20:5	750.8	782.1
	18:1/20:5	762.9	793.9
	15:0/22:6	749.1	779.9
	16:1/22:6	761.0	792.1
	16:0/22:6	763.0	793.9

*Fatty acyl chains are abbreviated as follows: 15:0, pentadecanoyl; 16:1, hexadecenoyl; 16:0, hexadecanoyl; 17:1, cyclopropane-containing hexadecanoyl; 17:0, heptadecanoyl; 18:1, octadecenoyl; 20:5, eicosapentaenoyl; 22:6, docosahexaenoyl.*

**FIGURE 2:**
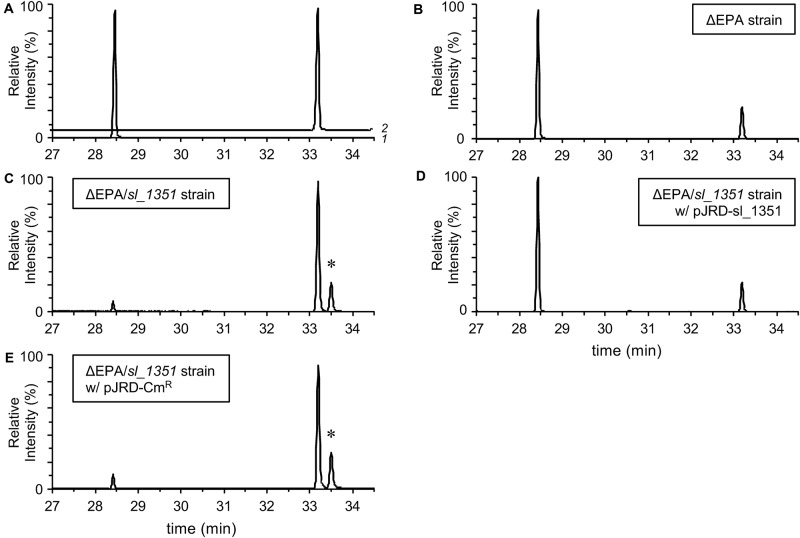
GC/MS analyses of fatty acid compositions. Authentic EPA and DHA methyl esters (**A**; traces 1 and 2, respectively) and the FAMEs of the ΔEPA strain **(B)**, ΔEPA/*sl_1351* strain **(C)**, ΔEPA/*sl_1351* strain harboring pJRD-sl_1351 **(D)**, and ΔEPA/*sl_1351* strain harboring pJRD-Cm^r^ **(E)** grown in the presence of DHA were analyzed. An asterisk indicates the peak putatively representing methyl docosaheptaenoate.

To confirm the occurrence of EPA in the ΔEPA cells, fatty acyl moieties of the extracted PLs were methyl-esterified and subjected to GC/MS analysis. As a result, EPA and DHA methyl esters, which eluted at 28.5 min and 33.2 min, respectively (Figure 2A), were detected in the FAMEs of the DHA-supplemented cells (Figure 2B) and accounted for 6.0 ± 0.3% and 1.8 ± 0.1% of the total FAMEs, respectively. Therefore, EPA production in the DHA-supplemented ΔEPA cells was unambiguously demonstrated and strongly supports the presence of a metabolic pathway that converts DHA into EPA. To estimate the rate of conversion from DHA to EPA, the bacterial culture was subjected to FAME analysis. The peak area for EPA methyl ester (corresponding to EPA produced) was divided by the sum of the peak area for EPA and DHA methyl esters (corresponding to DHA added), resulting in the conversion rate of 89.2 ± 0.6%.

### Mutagenesis Analysis of the Conversion Mechanism

**FIGURE 3:**
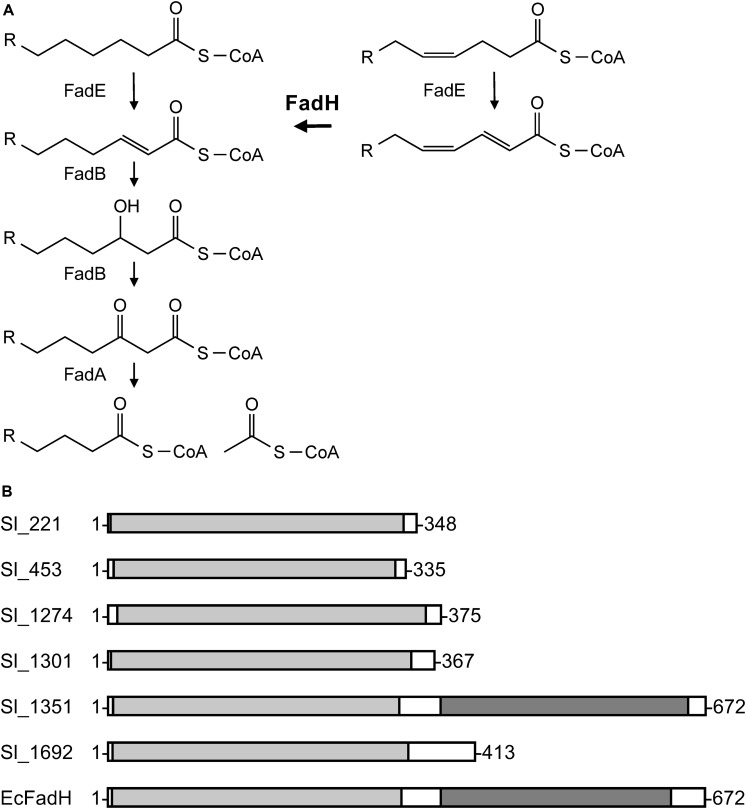
General β-oxidation reactions **(A)** and the domain structures of the six FadH homologs **(B)**. **(A)** FadE, acyl-CoA dehydrogenase; FadB, enoyl-CoA hydratase/3-hydroxyacyl-CoA dehydrogenase; FadA, 3-ketoacyl-CoA thiolase; and FadH, 2,4-dienoyl-CoA reductase. **(B)** The image was generated on the MOTIF Search website (https://www.genome.jp/tools/motif/) and modified. The light gray represents a catalytic domain and the dark gray NAD(P)H-binding domain. EcFadH represents FadH from *E. coli*.

As two carbon units and one double bond are eliminated when EPA is formed from DHA, we presumed that the conversion is mediated through the β-oxidation pathway or a similar metabolic route. As depicted in Figure 3A, the typical bacterial β-oxidation reactions for saturated fatty acids are achieved by the actions of three proteins with four enzymatic activities, acyl-CoA dehydrogenase (FadE), 2-enoyl-CoA hydratase/3-hydroxyacyl-CoA dehydrogenase (FadB), and 3-ketoacyl-CoA thiolase (FadA) (DiRusso et al., 1999). Unsaturated fatty acids with a double bond at odd-numbered carbon atoms are likewise degraded. On the other hand, the auxiliary enzyme 2,4-dienoyl-CoA reductase (FadH) is also required to metabolize unsaturated fatty acids, such as DHA, that have a double bond at even-numbered carbon atoms (You et al., 1989). Therefore, to investigate the conversion mechanism in *S. livingstonensis* Ac10, we examined the possible involvement of *fadH* genes by mutagenesis.

A homology search utilizing the amino acid sequence of a well-studied FadH from *E. coli* (Dommes and Kunau, 1984; Liang et al., 2000; Hubbard et al., 2003; Tu et al., 2008) as a query revealed that *S. livingstonensis* Ac10 has six FadH homologs. The homolog Sl_1351 has an N-terminal catalytic domain and C-terminal NAD(P)H-binding domain as with *E. coli* FadH, whereas the other five homologs (Sl_221, Sl_453, Sl_1274, Sl_1301, and Sl_1692) are composed of the catalytic domain only (Figure 3B). We disrupted each of the six *fadH* genes of the ΔEPA strain, which were confirmed by the formation of a PCR product in diagnostic PCR (data not shown), and analyzed the PL profiles of the mutants grown in the presence of DHA. ESI-MS analysis of the extracted lipids showed that EPA-containing PL levels markedly decreased in the ΔEPA/*sl_1351* strain (Figure 1C). Disruption of the other five genes had no significant impact on EPA-containing PL levels (Supplementary Figure S2). GC/MS analysis of FAMEs also showed a decrease in EPA levels in the ΔEPA/*sl_1351* strain (Figure 2C) and the conversion rate dropped to 12.3 ± 0.9%, which was 7.4-fold lower than that of the ΔEPA strain. For gene complementation, we introduced a plasmid (i.e., pJRD-sl_1351) that carries *sl_1351* gene into the ΔEPA/*sl_1351* mutant, which was confirmed through diagnostic PCR (data not shown). The conversion ability was restored to a level comparable to that of the ΔEPA strain by complementation with pJRD-sl_1351 (Figures 1D, 2D) but not with an empty vector (Figures 1E, 2E). In addition, an unknown compound that eluted at 33.5 min, close to the time at which DHA methyl ester eluted, was present in the FAMEs of the conversion-deficient cells (Figures 2C,E, asterisk) and showed a mass spectrum almost identical to that of DHA methyl ester, with slight differences (data not shown). The peak probably represents the 2,4-dienoyl-CoA derivative of DHA (docosa-2,4,7,10,13,16,19-heptaenoyl-CoA), which can accumulate due to deficiency in the conversion. Although a small amount of EPA was still produced in the ΔEPA/*sl_1351* mutant, these results strongly suggested that Sl_1351 plays a key role in DHA conversion.

### *In vitro* Assay of the Recombinant Sl_1351 Protein

**FIGURE 4:**
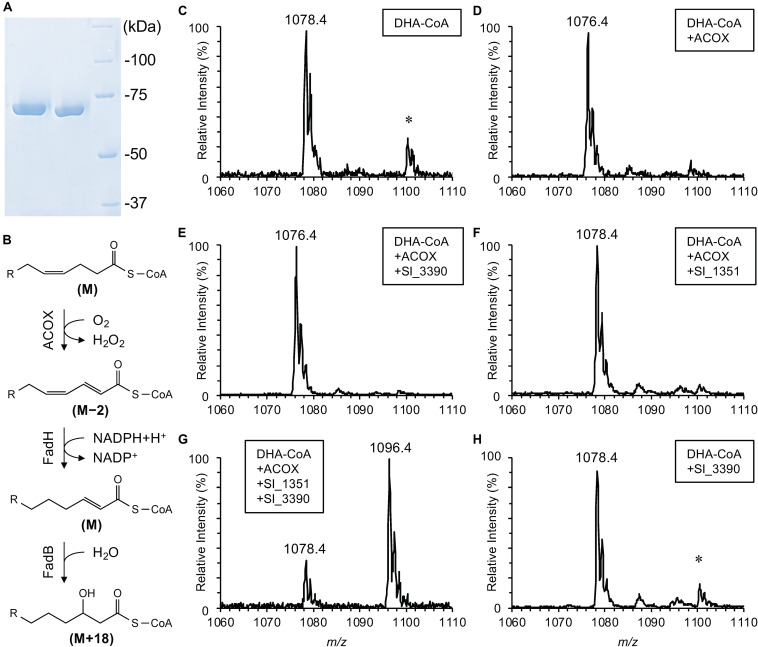
*In vitro* assay for recombinant Sl_1351. SDS-PAGE analysis of the purified recombinant proteins (**A**; lane 1, Sl_3390; lane 2, Sl_1351; lane 3, protein standards), a reaction scheme of the β-oxidation (**B**; relative mass changes are indicated in the brackets), and ESI-MS analyses of DHA-CoA **(C)** and its derivatives after incubation with ACOX alone **(D)**, ACOX and Sl_3390 **(E)**, ACOX and Sl_1351 **(F)**, ACOX, Sl_1351 and Sl_3390 **(G)**, and Sl_3390 alone **(H)**. The *m/z* values are provided for [M + H]^+^ ions. The asterisk indicates [M + Na]^+^ ions with *m/z* larger than the corresponding [M + H]^+^ ions by 22.

To further examine the role of Sl_1351 in DHA conversion, the recombinant Sl_1351 protein was heterologously produced in *E. coli* BL21(DE3) and purified by affinity chromatography (Figure 4A). The recombinant Sl_3390 protein, a putative FadB of *S. livingstonensis* Ac10, was also prepared in the same way and was found to have 2-enoyl-CoA hydratase, but not 3-hydroxyacyl-CoA dehydrogenase, activity (Supplementary Figure S3), most likely due to product inhibition in the absence of FadA (Binstock and Schulz, 1981). For the 2,4-dienoyl-CoA reductase assay, the substrate docosa-2,4,7,10,13,16,19-heptaenoyl-CoA was prepared from DHA-CoA by using ACOX, and the production of docosa-2,7,10,13,16,19-hexaenoyl-CoA was validated by using the recombinant Sl_3390 (Figure 4B). DHA-CoA was incubated with the three proteins ACOX, Sl_1351, and Sl_3390, and the products were analyzed by ESI-MS.

Without the addition of the enzymes, DHA-CoA was detected primarily as an [M + H]^+^ ion with an *m/z* of 1078.4 (Figure 4C). This value was in good agreement with the theoretical monoisotopic mass of DHA-CoA, 1078.3522. The addition of ACOX decreased the *m/z* value by 2 (Figure 4D), indicating the removal of two hydrogen atoms to form docosaheptaenoyl-CoA (Figure 4B, step 1). As expected, the addition of both ACOX and Sl_3390 did not cause hydration of the product (Figure 4E). When DHA-CoA was incubated with both ACOX and Sl_1351, an ion with an *m/z* of 1078.4 was detected (Figure 4F), which likely represents the reduced product, docosa-2,7,10,13,16,19-hexaenoyl-CoA (Figure 4B, step 2). The *m*/*z* value of this ion is indistinguishable from the [M + H]^+^ ion of DHA-CoA (Figure 4C). However, the product served as the substrate of Sl_3390 and was hydrated with the *m/z* value increasing by 18 in the presence of all the three enzymes (Figure 4B, step 3 and Figure 4G), whereas DHA-CoA was not directly hydrated by Sl_3390 (Figure 4H). Therefore, the product of Sl_1351 would be docosa-2,7,10,13,16,19-hexaenoyl-CoA, a typical β-oxidation intermediate. Altogether, these data suggested that *sl_1351* codes for 2,4-dienoyl-CoA reductase required to metabolize DHA and support the results of the mutagenesis experiments.

### The Effects of DHA Supplementation on the Cold-Sensitive Phenotypes of the ΔEPA Strain

EPA and DHA are long carbon chain compounds (20 and 22 carbons, respectively) that are multiply unsaturated (five and six double bonds, respectively) and categorized into the same class of ω-3 long chain PUFAs. Nevertheless, some marine bacteria mainly produce EPA, whereas others mainly DHA (DeLong and Yayanos, 1986; Yazawa, 1996; Nogi et al., 1998; Kawamoto et al., 2009; Hashimoto et al., 2015), suggesting that the bacteria have an obligate requirement for either of these PUFAs. Therefore, we considered whether DHA acts as a substitute for EPA in *S. livingstonensis* Ac10. In our previous study, supplementation with DHA-containing PLs suppressed growth defects of the ΔEPA strain; however, it remained unclear whether DHA itself substituted for EPA, because EPA was formed from supplemented DHA through the conversion pathway (Sato et al., 2008). As conversion was largely suppressed by the disruption of the *sl_1351* gene, we reassessed the interchangeability between DHA and EPA using the ΔEPA/*sl_1351* strain.

**FIGURE 5:**
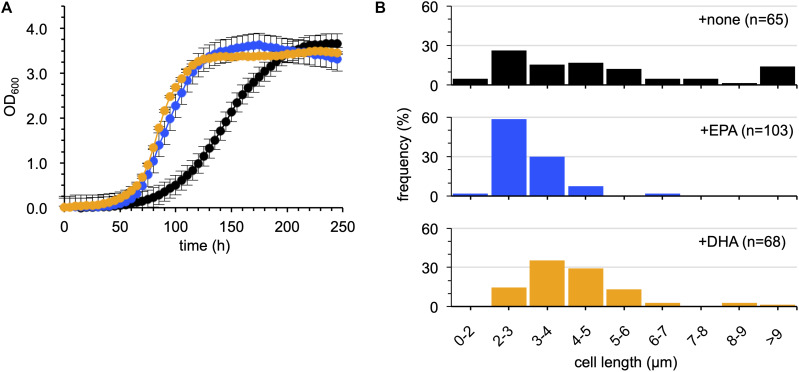
Examination of the effects of EPA and DHA supplementation on the growth of the ΔEPA/*sl_1351* strain. The growth **(A)** and cell length distribution **(B)** of non-supplemented (black), EPA-supplemented (blue), and DHA-supplemented (orange) cells are shown. The growth curves were produced according to three independent experiments. The numbers of cells subjected to size measurements are indicated in brackets in each graph.

The rationale of the assessment was that if DHA is biologically equivalent to EPA, DHA should restore the cold-sensitive phenotypes of EPA-less mutants, such as growth retardation and filamentous cell formation at 4°C, as EPA did (Tokunaga et al., 2017) in the absence of Sl_1351. As shown in Figure 5A, supplementation with DHA suppressed the growth retardation of the ΔEPA/*sl_1351* strain as much as EPA supplementation. DHA supplementation also suppressed filamentation of the cells (Figure 5B). However, the DHA-supplemented cells were longer than the EPA-supplemented cells. At OD_600_ of approximately 1.0, 90% of the EPA-supplemented cells reverted to the normal cell length of 2–4 μm (Figure 5B), and the average length was 3.0 ± 0.8 μm, which was shorter than that of the non-supplemented cells at 5.5 ± 4.3 μm. On the other hand, only half of the DHA-supplemented cells were 2–4 μm long, and 43% showed slightly longer lengths of 4–6 μm (the average length, 4.3 ± 1.7 μm). Therefore, although DHA is beneficial to the growth of *S. livingstonensis* Ac10 at cold temperatures, it is not biologically equivalent to EPA.

## Discussion

We previously found that the EPA-producing bacterium *S. livingstonensis* Ac10 utilized exogenous DHA, added to the culture medium as an acyl group of PL, to produce EPA (Sato et al., 2008). Watanabe et al. (1994) reported the uptake of exogenous DHA by several bacterial species, among which *E. coli* was found to convert exogenous DHA into EPA. However, the molecular mechanism for the conversion remained to be elucidated. Here, we revealed the conversion mechanisms focusing on the putative *fadH* genes. We demonstrated through mutagenesis studies that disruption of the *sl_1351* gene markedly suppressed conversion (Figures 1, 2), and through an *in vitro* enzymatic assay that the recombinant Sl_1351 protein had 2,4-dienoyl-CoA reductase activity toward the 2,4-dienoyl-CoA derivative of DHA (Figure 4). These data strongly suggest that Sl_1351 is a key enzyme in the conversion of DHA to EPA. However, there remained decreased level of conversion in the *sl_1351*-disrupted cells. This can be explained by the subtle hydratase activity of FadB toward docosa-2,4,7,10,13,16,19-heptaenoyl-CoA, as Yang et al. (1986) reported that the 2,4-dienoyl-CoA intermediate can be a substrate of FadB and undergo β-oxidation cycles, although the reactions are thermodynamically unfavorable. Additionally, minor contributions of the other five FadH homologs are possible.

As Sl_1351 is most homologous to *E. coli* FadH (56.5% identical) of the six FadH homologs of *S. livingstonensis* Ac10, and has both N-terminal catalytic and C-terminal NAD(P)H-binding domains (Figure 3), it is likely that Sl_1351 is a *bona fide* FadH and, thus, the conversion is mediated through a typical β-oxidation pathway. β-oxidation enzymes catalyze repeated cycles of reactions to eliminate two carbon units as a form of acetyl-CoA at the end of each cycle and, thus, can sequentially degrade DHA to EPA and further to octadeca-3,6,9,12,15-pentaenoic acid and hexadeca-4,7,10,13-tetraenoic acid. However, such PUFAs were undetectable in our ESI-MS and GC/MS analyses (data not shown). No PUFAs other than EPA were observed when the cells were supplemented with a synthetic DHA-containing PL (Sato et al., 2008). These observations imply that *S. livingstonensis* Ac10 preferentially produces EPA from DHA without metabolizing it further and forms EPA-containing PLs, which are important membrane constituents for the bacterium to survive in cold environments (Kawamoto et al., 2009).

Further investigations could determine how the β-oxidation pathway is ceased after one cycle of reactions to preferentially generate EPA. This regulation is exemplified by the Sprecher pathway used for DHA biosynthesis in mammals. The pathway involves initial production of tetracosa-6,9,12,15,18,21-hexaenoic acid from α-linolenic acid via an elongase-desaturase system and the following retroconversion to DHA through a single cycle of β-oxidation reactions (Voss et al., 1991). Peroxisomal β-oxidation enzymes are responsible for the chain-shortening step (Su et al., 2001); however, the exact mechanism remains enigmatic. One possible mechanism for the selective conversion of DHA to EPA by *S. livingstonensis* Ac10 is that the FadE enzyme, which catalyzes the first committed step of the β-oxidation pathway, is active toward DHA-CoA but not, or to a lesser extent, toward EPA-CoA. If this is the case, the next cycle of the β-oxidation reaction would not proceed efficiently, and EPA-CoA is preferentially used by PlsC as the substrate for PL formation (Ogawa et al., 2018). *S. livingstonensis* Ac10 has two FadE homologs that are highly homologous to *E. coli* FadE, suggesting that at least one of them is active toward DHA-CoA. To examine whether FadE is a key regulator in the conversion of DHA to EPA, we are currently analyzing the *in vivo* and *in vitro* characteristics of the FadE homologs.

Our data shown in Figure 5 imply that DHA partially substitutes for EPA but is not interchangeable. Given that the abnormal cell elongation of the ΔEPA strain is presumably caused by the deficiency in cell division (Kawamoto et al., 2009), the distinct cell length distribution between the EPA- and DHA-supplemented cells suggests that EPA is more favorably exploited for this process. Although the exact function of PUFAs in marine bacteria remains unknown, their hyperflexible polyene hydrocarbon chain is considered to be a key factor, which can render lipid membrane more fluid and flexible and solvate a membrane protein more efficiently than saturated and monounsaturated fatty acids (Antonny et al., 2015; Yoshida et al., 2016). Interestingly, previous studies utilizing a model eukaryotic membrane have suggested that EPA and DHA, in the form of free acid or an acyl chain of PLs, are moderately different from one another in their physicochemical properties such as the degree of conformational changes within membranes and (dis)ordering lipid bilayer, and, thus, potentially have a distinct impact on cellular events (Williams et al., 2012; Leng et al., 2018; Sherratt and Mason, 2018). As cell division involves the dynamic rearrangement of cellular membranes and is achieved by a variety of membrane proteins such as those forming divisome complex, we speculate that it proceeds more efficiently in the presence of membrane PLs containing EPA than those containing DHA due to the difference in their physicochemical properties. Detailed comparative studies regarding EPA and DHA are necessary to clarify their distinctive physiological functions, and the conversion-deficient strain constructed here will contribute to those studies.

## Data Availability Statement

The datasets generated for this study can be found in the DDBJ LC528929, LC528930, LC528931, LC528932, LC528933, LC528934, LC528935.

## Author Contributions

TO, JK, and TK designed the study. KH and YY performed the experiments. TO and TK wrote the manuscript. All authors discussed the results.

## Conflict of Interest

The authors declare that the research was conducted in the absence of any commercial or financial relationships that could be construed as a potential conflict of interest.

## Footnotes

1 https://imagej.nih.gov/ij/

2 https://www.fruitfly.org/seq_tools/promoter.html

## Abbreviations

ACOX: acyl-CoA oxidase
CoA: coenzyme A
Cm: chloramphenicol
DHA: docosahexaenoic acid
EPA: eicosapentaenoic acid
ESI: electrospray ionization
FAME: fatty acid methyl ester
GC: gas chromatography
HEPES: *N*-2-hydroxyethylpiperazine-*N*′-2-ethanesulfonic acid
Km: kanamycin
MS: mass spectrometry
PL: phospholipid
PUFA: polyunsaturated fatty acid
Rf: rifampicin
Ura: uracil.
