# Supplementary figures and images for: Bioconversion From Docosahexaenoic Acid to Eicosapentaenoic Acid in the Marine Bacterium Shewanella livingstonensis Ac10
Source: Front Microbiol. 2020 May 26;11:1104. doi: 10.3389/fmicb.2020.01104 (PMC7264947; doi:10.3389/fmicb.2020.01104)

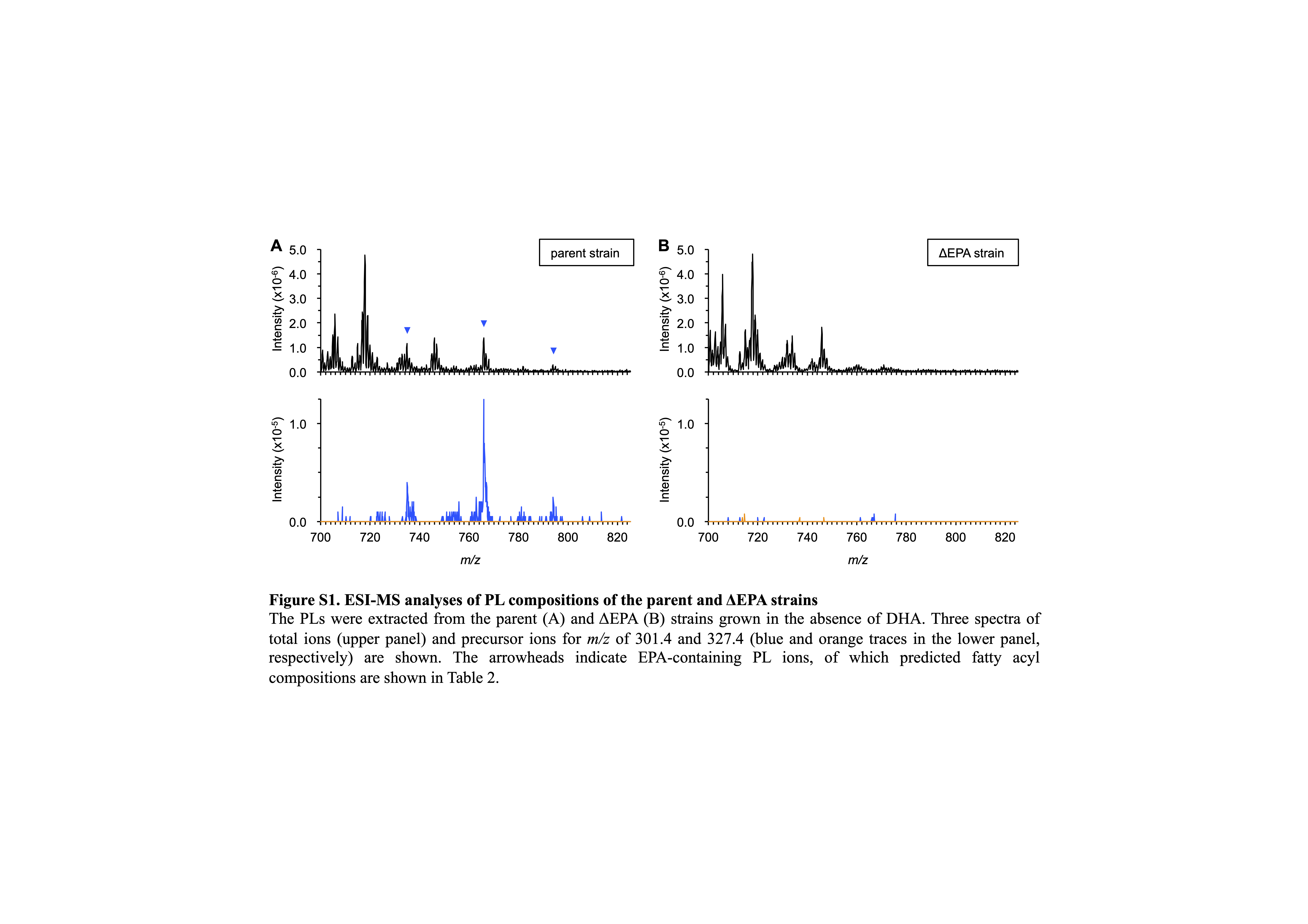

Supplement: Supplementary file 1 [file Image_1.tiff]

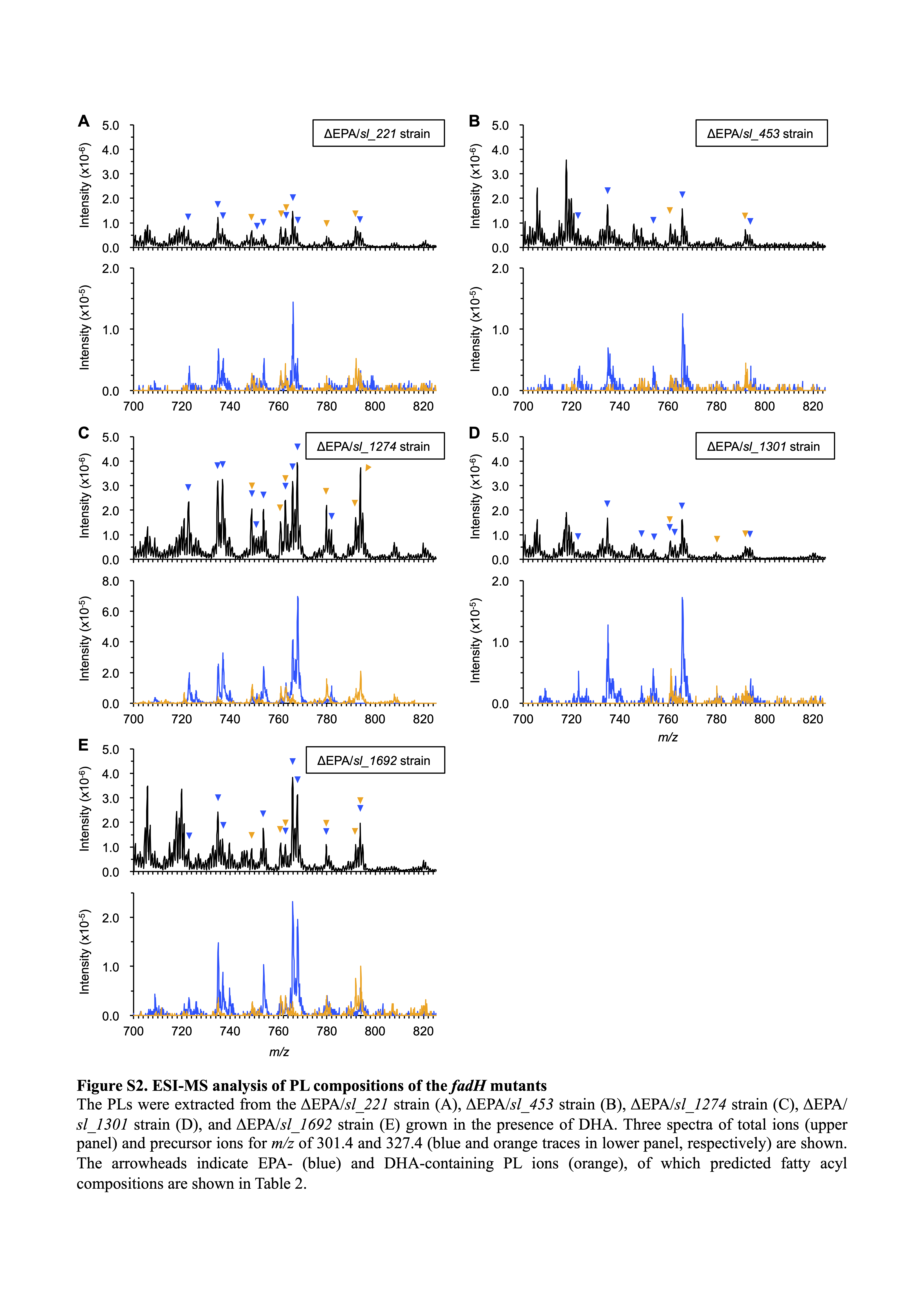

Supplement: Supplementary file 2 [file Image_2.tiff]
